# Population and Functional Genomics of Neisseria Revealed with Gene-by-Gene Approaches

**DOI:** 10.1128/JCM.00301-16

**Published:** 2016-07-25

**Authors:** Martin C. J. Maiden, Odile B. Harrison

**Affiliations:** Department of Zoology, University of Oxford, Oxford, United Kingdom; Emory University

## Abstract

Rapid low-cost whole-genome sequencing (WGS) is revolutionizing microbiology; however, complementary advances in accessible, reproducible, and rapid analysis techniques are required to realize the potential of these data. Here, investigations of the genus Neisseria illustrated the gene-by-gene conceptual approach to the organization and analysis of WGS data. Using the gene and its link to phenotype as a starting point, the BIGSdb database, which powers the PubMLST databases, enables the assembly of large open-access collections of annotated genomes that provide insight into the evolution of the Neisseria, the epidemiology of meningococcal and gonococcal disease, and mechanisms of Neisseria pathogenicity.

## INTRODUCTION

Using culture techniques, microscopy, and animal models, Robert Koch and his contemporaries established the paradigm that bacteria could be classified into discrete groups which consistently exhibited distinct properties. Although this was first demonstrated in bacterial pathogens, such groupings are found in all bacterial populations and communities; however, precise and universal “species definitions” remain elusive, even after more than 140 years. Recent advances in DNA sequence analysis have generated large volumes of data that support the concept of structure within bacterial populations, but these data have also indicated why accurate species definitions remain difficult to attain. The ability to determine nearly complete drafts or “whole-genome sequences” (WGSs) of bacterial genomes rapidly and inexpensively has been foremost in these advances (1).

We now know that bacterial populations have existed for around 3.5 billion years and are extraordinarily diverse in terms of gene content, nucleotide sequence, and organization. This diversity has been generated by (i) the cumulative effects of mutation over time; (ii) intragenome rearrangement and reorganization; and (iii) horizontal gene transfer (HGT) among bacteria that do not share an immediate common ancestor. The limits of HGT can be extremely wide, enabling bacteria to recruit genetic variation from evolutionarily highly divergent sources, including other domains of life, providing a “gene pool” of bewildering variety. Most bacteria have “open genomes” that comprise “core genes,” those genes present in most or all members of a particular group, and “accessory genes,” which are variably present within that group. Combined, these represent a “pan-genome” representing all of the genes available to a given group of bacteria.

Now that WGS data collection is rapid and inexpensive, the challenge is to catalogue bacterial diversity and link it to information relating to an organism's phenotype, i.e., what it does, and its provenance, i.e., where it comes from. Within PubMLST.org, open-access, Web-based databases address this problem using a gene-by-gene approach, facilitated by the bacterial isolate genome sequence database (BIGSdb) software (2). Using this approach, bacterial species can be rapidly identified, virulence factors can be detected, outbreaks can be recognized, and antimicrobial resistance (AMR) genotypes can be obtained.

## THE BACTERIAL ISOLATE GENOME SEQUENCE DATABASE (BIGSdb)

BIGSdb links three types of information: (i) provenance and phenotype data (“metadata”); (ii) sequence data, which can be anything from a single gene sequence to a complete closed genome; and (iii) an expanding catalogue of loci, identifying specific regions of the genome and their genetic variations. This philosophy can be thought of as a whole-genome (wg) approach to multilocus sequence typing (MLST) (3, 4), or “wgMLST,” and it permits rapid, scalable, flexible storage and analysis of data (5).

BIGSdb stores isolate records, including provenance and phenotype data, linked to “sequence bins” that can contain any assembled WGS data available for the isolate. These are hyperlinked to the unassembled raw data from which the compiled sequences were derived, which are stored in repositories such as the European Nucleotide Archive (ENA) at the European Bioinformatics Institute (EBI) or the Sequence Read Archive (SRA) at the National Center for Biotechnology Information (NCBI). Within BIGSdb, tables of known allele sequences are maintained for each locus that has been defined, and when new sequence data are submitted to the database, a search algorithm (currently Blast) is used to identify known loci and variants. If a known sequence is detected, it is “tagged” in the corresponding sequence bin for ease of later identification and the allele number for that sequence is associated with the isolate record. If it is an unknown variant of a known allele, the sequence is marked for curator verification and, if appropriate, a novel allele number is assigned. This iterative process continually builds an expanding catalogue of the known diversity of all defined loci in the database (5). Additional genes are identified in unannotated areas of WGS data using gene-finding software such as Prokka, expanding the catalogue of defined genes in the database. Ultimately, this process will lead to a complete organized and curated catalogue of the pan-genome of any organism (6).

Gene-based comparative genomics is possible within BIGSdb using the Genome Comparator tool, which compares groups of shared genes among isolates with any number of loci predefined in the database or in an annotated reference genome. For each locus, the allele sequences, designated by integers, are compared and used to generate a distance matrix that is based on the number of variable loci across the genome. The distance matrices can be resolved in a number of ways, including as two-dimensional graphs using the NeighborNet algorithm. The Genome Comparator output simultaneously provides lists of loci that are identical, variable, missing, or incomplete (only partially present in the sequence bin, usually because of incomplete assembly) among data sets, rapidly resolving bacterial population relationships and elucidating loci core to a particular data set (7).

An example of this approach is the use of ribosomal MLST (rMLST) to define species groupings and strain types (8–11). A pan-domain database, built within PubMLST (www.pubmlst.org/rmlst), catalogues variation in the ribosomal protein subunit genes, comprising those encoding the proteins in the small (*rps*) and large (*rpl*) ribosome protein subunits. At the time of writing, this database included more than 130,000 sets of assembled WGS data for many diverse bacteria obtained from publicly available sources. The rMLST genes represent a defined set of core genes, present in all bacteria, which provide the basis for an efficient and rapid identification and characterization scheme for bacterial typing and taxonomy from “domain to strain.”

**FIG 1:**
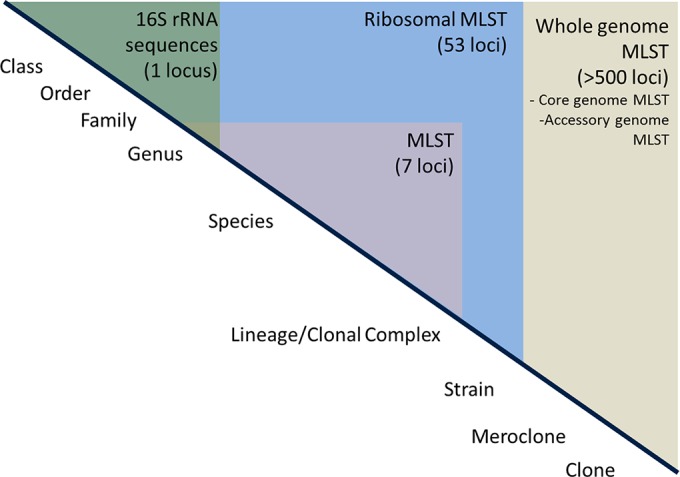
Hierarchical approach to WGS data analysis. The data represent the increasing resolution seen in analyzing different and increasing numbers of loci in bacterial genomes (above the diagonal line, shaded), along with their relationship to nomenclature (below the line). 16S rRNA efficiently identifies bacteria to the genus level, whereas conventional locus MLST (sometimes called multilocus sequence analysis [MLSA]) enables resolution within genera and species. The 53-locus rMLST approach allows species identification and resolution within species, while the highest levels of resolution are obtained with whole, core, and/or accessory genome MLST. (Image republished from reference 5 with permission of the publisher.)

Through the use of this hierarchical gene-by gene approach to genome analysis, any combination of genes can be compared among isolates, ranging from the comparison of single loci to analyses of whole genomes, resulting in a genome-wide MLST profile, i.e., wgMLST (Fig. 1) (5). Few isolates, however, share all loci, so comparisons of data from a defined core set of loci (cgMLST) provide high-resolution analyses among members of a group of related but not identical isolates. The robustness of these processes to generate data is at least as reliable as the robustness of conventional sequencing technologies and has been validated through the analysis of a defined Neisseria meningitidis isolate collection (7).

## GENOMICS AND THE NEISSERIA

The genus Neisseria is a group of Gram-negative, oxidase-positive aerobic Betaproteobacteria, which are commonly associated with the dental and mucosal surfaces of animals and humans (12). Most of these organisms are harmless members of the commensal microbiota; however, the genus contains two important pathogens: N. meningitidis, the meningococcus; and Neisseria gonorrhoeae, the gonococcus. The meningococcus is often referred to as an “accidental pathogen,” as the bacterium predominantly resides as a harmless commensal in the adult human nasopharynx, rarely becoming invasive. Disease is an evolutionary dead end for the meningococcus, as it does not usually lead to onward transmission (11). In contrast, the gonococcus is an obligate human pathogen, with colonization usually resulting in a localized inflammatory response. N. gonorrhoeae infections can have severe sequelae, ranging from disseminated infection to salpingitis or pelvic inflammatory disease, with gonococcal infection asymptomatic in over 95% of women and a significant cause of infertility in this patient group. Emerging antibiotic resistance of the gonococcus has become a major global health challenge.

**FIG 2:**
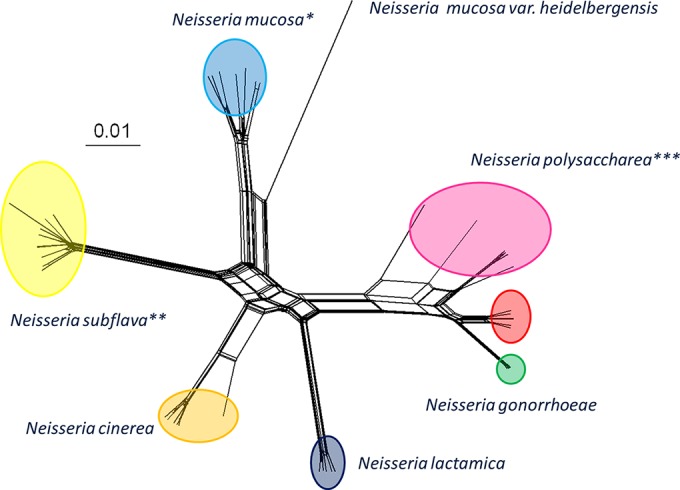
Evolutionary relationships among Neisseria species based on concatenated sequences of the 53 ribosomal gene proteins (rMLST). The relationships among different Neisseria species were reconstructed with nucleotide sequences from 49 ribosomal gene proteins. Single asterisks (*) denote a cluster of Neisseria mucosa species in which isolates previously identified as being Neisseria sicca and Neisseria macacae species were found, indicating that rMLST analysis had identified these as being variants of the N. mucosa species. Double asterisks (**) denote a cluster composed of Neisseria subflava species in which isolates previously identified as Neisseria flavescens had clustered. Triple asterisks (***) denote a polyphyletic group comprising N. polysaccharea isolates indicative of the presence of more than one N. polysaccharea species. (Image republished from reference 6with permission of the publisher.)

Despite these dramatic phenotypic differences, the relationships among members of the Neisseria genus are often poorly resolved using conventional typing and/or molecular approaches such as 16S rRNA sequencing. This poor species resolution, which Neisseria species share with the members of many other bacterial genera, is a consequence of shared ancestry combined with extensive HGT (8). Relationships among Neisseria species can, however, be resolved using cgMLST and rMLST methods. A total of 246 genes consisting of 190,534 nucleotides and amounting to 8.68% of the Neisseria genome have been identified as core to the genus (Neisseria genus cgMLST) (8). Phylogenetic analyses of these core genes in a collection of isolates representative of the genus identified seven distinct Neisseria groups that were congruent with rMLST analyses. These analyses demonstrated, for example, that Neisseria polysaccharea isolates formed a polyphyly, with isolates descendant from two or more ancestral groups, something which hitherto had not been apparent using conventional phenotypic and 16S rRNA sequence analyses (Fig. 2) (8).

The greater resolution afforded by rMLST and cgMLST was further demonstrated through comparisons of data from a newly described Neisseria species, Neisseria oralis (13). Combined analyses, including analyses of 16S rRNA and 23S rRNA gene sequence similarity, DNA-DNA hybridization, and cellular fatty acid analysis, along with phenotypic analyses indicated that this novel species was closely related to the commensal Neisseria lactamica, which was consistent with the presence of β-galactosidase in these isolates, a trait considered to be diagnostic of N. lactamica (14). Phylogenetic reconstructions using rMLST and cgMLST analysis of the 246 genes core to the Neisseria genus, however, demonstrated that these isolates were in fact monophyletic with isolates previously named “Neisseria mucosa var. heidelbergensis” (15), thereby enabling the consolidation of N. oralis and “N. mucosa var. heidelbergensis” into a single species group using the published name N. oralis. That study demonstrated how reliance on biochemical tests to differentiate N. lactamica from other Neisseria species may be unreliable, given that N. oralis was found to contain intact lactose fermentation *lacY* and *lacZ* genes indicative of β-galactosidase activity.

In all of those cases, nucleotide sequence data from multiple loci were readily available from WGS data; however, such information is not always economically or practically available from all specimens, especially in resource-poor settings. Therefore, the PubMLST.org/neisseria resource was employed to develop a new assay that met these requirements. This assay targets a 413-bp fragment of the 50S ribosomal protein L6 (*rplF*) gene, which is highly diagnostic of membership of Neisseria species. To develop the assay, phylogenies were reconstructed from multiple *rpl* and *rps* loci and from WGS data and were compared; the results showed that the clustering of the *rlpF* fragments was highly congruent with clusters obtained from concatenated rMLST sequences and cgMLST sequences (16). In a collection of over 900 isolates, no *rplF* alleles were shared among commensals and pathogens or between meningococci and gonococci, confirming the suitability of the *rplF* fragment assay in differentiating pathogenic and commensal Neisseria species. This assay was developed and validated over a matter of a few weeks and proved invaluable in the MenAfriCar study, which required rapid and inexpensive species identification of thousands of samples collected in the African meningitis belt (17).

## EVOLUTION OF THE NEISSERIA

At the time of writing, high-quality WGS “draft genomes” existed for over 7,000 Neisseria isolates and, while the majority of these were from N. meningitidis and N. gonorrhoeae, at least one representative isolate from each known Neisseria species was available. Analyses of these data from representative isolates demonstrated that their genomes are similar with respect to overall composition, size, and architecture but that extensive diversity is present at the nucleotide level among the species (18). For example, comparison of the finished genomes from the closely related N. lactamica, N. meningitidis, and N. gonorrhoeae species identified a core genome constituting approximately 60% of all coding sequences (CDSs) indicative of shared ancestry (18). Nonetheless, it was possible to identify sequence polymorphisms specific to each species, which was consistent with infrequent interspecies genetic exchange and independent evolution of the core genome and/or with stabilizing selection favoring species-specific allelic variation.

In contrast, the Neisseria accessory genome has been found to comprise a variable number of loci that are not uniformly distributed among all Neisseria species and includes intra- and extrachromosomal elements such as plasmids, genetic islands (GIs), and prophages (12). It was initially thought likely that the accessory genome included the virulence determinants necessary for a meningococcus or gonococcus to become invasive; however, it has become increasingly clear that many such “virulence loci” frequently move among species, whether pathogenic or not, by HGT (19). This shows that a “pathogenome” composed of accessory genes is not easily defined; indeed, many of the putative loci associated with virulence have been found in commensal Neisseria species, indicating that differences in sequence variation and gene expression may be the determining factors eliciting the distinct phenotypes of Neisseria species (20).

## MOLECULAR EPIDEMIOLOGY AND POPULATION STRUCTURE

Despite extensive HGT, a readily discernible structure exists in meningococcal populations, first identified by multilocus enzyme electrophoresis (MLEE) and later with MLST (4) and rMLST (10). Conventional MLST schemes index variation in seven “MLST loci,” with each unique sequence for each locus generating an allelic profile or a sequence type (ST). Using ST designations, isolates are grouped into clonal complexes (ccs) which correspond to lineages. The ccs are defined as groups of STs sharing at least four of the seven loci in common with a central ST which gives the cc its name, e.g., the “ST-11 clonal complex,” or cc11 (4).

The establishment of N. meningitidis ccs has been instrumental in improving our understanding of the epidemiology of meningococcal disease and enhancing surveillance (4). For example, the EU-MenNet project, which analyzed over 4,000 disease-associated N. meningitidis isolates from 18 European countries from 2000 to 2002, found a predominance of hyperinvasive ccs, the most prevalent being cc41/44, cc11, cc32, cc8, and cc269 (21). Further, population genetic studies have consistently found that a limited number of hyperinvasive lineages caused most meningococcal invasive disease cases, with carriage isolates being more diverse and belonging to thousands of lineages, many of which have never been associated with disease.

In addition to MLST, cataloguing of variable regions within the antigen-encoding genes *porA* and *fetA* enhances meningococcal classification. Combined, these data generate a strain designation profile consisting of the following serogroups (capsular polysaccharides): PorA type; FetA type; sequence type (clonal complex) (e.g., B: P1.19,15: F5-1: ST-33 [cc32]). These data have been produced with conventional nucleotide sequencing methods, but automated extraction of such typing information can be rapidly obtained from WGS data deposited in BIGSdb, providing clinically useful strain characterization to physicians and public health professionals in a timely, concise, and understandable manner which is “backwards compatible” and which can inform public health interventions (22).

An important element in the biology of the meningococcus is its ability to live asymptomatically in the nasopharynx, with asymptomatic transmission common in the human population. Understanding the impacts of vaccination in carried meningococci is therefore essential to determine the effects of vaccination and whether herd immunity has been achieved. Large-scale carriage studies have been undertaken to survey meningococcal carriage pre- and postvaccination, including those conducted by the UKMenCar Consortium (23) and the African Meningococcal Carriage (MenAfriCar) Consortium. Established in 2008, MenAfriCar conducted carriage surveys across the meningitis belt during the introduction of the novel vaccine PsA-tt (MenAfriVac) (24). These identified a diverse meningococcal population and noted a significant increase in the detection of a particular unencapsulated meningococcus after vaccine introduction concomitant with a decrease in detection of serogroup A N. meningitidis isolates (17).

The same genes and gene fragments devised for N. meningitidis MLST have been used to study N. gonorrhoeae populations (25, 26) and can be effective in studying the long-term global epidemiology and evolution of N. gonorrhoeae (27). The use of seven-locus MLST alone, however, is insufficient to identify person-to-person spread, sexual networks, or emerging antimicrobial-resistant strains. In such circumstances, the N. gonorrhoeae multiantigen sequence typing (NG-MAST) scheme is used. This types variable internal fragments from two polymorphic genes, *porB* (encoding the major porin) and *tbpB* (encoding subunit B from the transferrin binding protein), and is highly discriminatory (28). NG-MAST has been successfully used: for tracing sexual contacts (29); to investigate treatment failures (30); as a tool for predicting specific antimicrobial resistance phenotypes (31, 32); and to study the global dissemination of NG-MAST ST-1407, which is associated with reduced susceptibility and resistance to third-generation cephalosporins (32).

## PATHOGENICITY

Much research into meningococci involves the investigation of putative virulence factors, but the main prerequisite for meningococcal invasion is the expression of an extracellular polysaccharide capsule. Of the 12 known capsules, as defined by their biochemical properties, only 6, corresponding to serogroups A, B, C, W, X, and Y, cause the majority of meningococcal disease. Further, particular hypervirulent clonal complexes are associated with the expression of certain capsules (33). The remaining meningococcal serogroups (H, E, L, I, K, and Z) are rarely associated with invasive disease but occur in asymptomatic carriage in the nasopharynx along with meningococci that cannot express a capsule as the capsule expression region is replaced by the null locus, *cnl*, which is also present in the acapsulate gonococcus. Interestingly, some meningococcal *cnl* genotypes can also cause invasive disease.

Protein-conjugate vaccines that target meningococcal polysaccharides are the most effective vaccines currently available; however, the selection pressures imposed by such vaccines potentially result in the emergence of virulent variants expressing nonvaccine capsules, as seen with Streptococcus pneumoniae. Cocolonization in the human nasopharynx of meningococci with different serogroups can lead to capsule switching generated by HGT, although, to date, this has not threatened meningococcal immunization programs. In the absence of fully comprehensive meningococcal vaccines, it remains important that the serogroups associated with meningococcal disease are monitored. All of the genes implicated in capsule biosynthesis are defined in the pubMLST.org/neisseria database along with other potential virulence determinants (6).

## MENINGOCOCCAL AND GONOCOCCAL EPIDEMIOLOGICAL SURVEILLANCE

**FIG 3:**
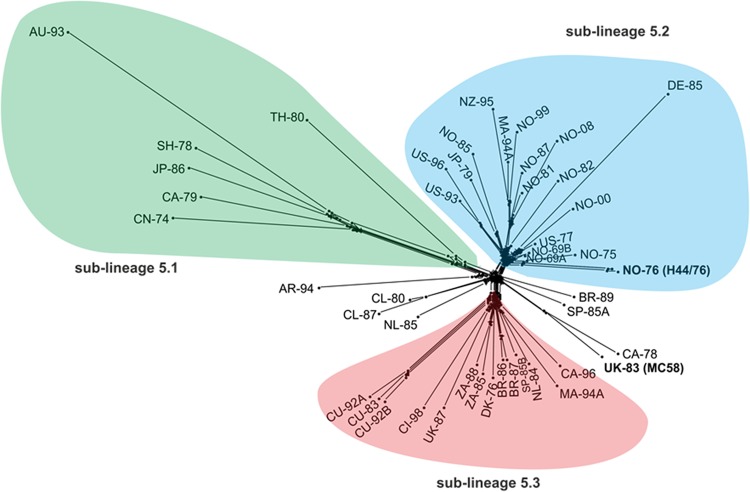
Core genome genealogical analysis of a global lineage 5 meningococcal population dating from 1969 through 2008. Lineage 5 meningococci (equivalent to the hyperinvasive ST-32 clonal complex identified by MLST) have been responsible for serogroup B meningococcal disease outbreaks globally for more than 30 years. In this analysis, a total of 1,752 loci (core to the lineage) were compared, identifying the presence of three distinct sublineages which were classified into the “Asian group” (sublineage 5.1), the “North European-Norwegian group” (sublineage 5.2) which contained isolates with PorA type P1.7,16, and a “Latin American group” (sublineage 5.3) with PorA type P1.19.15. (Image republished from reference 41 with permission of the publisher.)

For clinical and public health exploitation of WGS data, it is necessary to extract information that can place the isolate in context with information from other typing methods, thereby improving analysis of outbreaks and informing the implementation of control measures (11). Gene-by-gene analyses facilitate this approach, with several such studies successfully defining N. meningitidis outbreaks at the local (11), national (9, 34), and international (35) levels (Fig. 3). While meningococcal disease is a global phenomenon, the United Kingdom has a data set that is of particularly high quality due to its size and the fact that meningococcal disease has been reportable in England and Wales since 1912. There have been wide fluctuations in the incidence of disease in the subsequent years, with the most significant peaks occurring during both world wars (9), after which a substantial increase in disease was apparent from the early 1980s to the early 2000s. This included epidemics starting in the 1990s caused by hyperinvasive serogroup C cc11 meningococci (9).

Routine disease surveillance of meningococcal disease with WGS in the United Kingdom was initiated by the Meningitis Research Foundation Meningococcus Genome Library (MRF-MGL), which contains WGS data from all cases of invasive meningococcal disease that occurred in England and Wales dating from 2010 onwards (9). Gene-by-gene analysis of these data enables rapid and comprehensive meningococcal isolate characterization and was instrumental in identifying a rapid increase in serogroup W meningococcal disease caused by cc11 strains, with further WGS analysis indicating that this was a novel variant with an expanding global distribution (36). These observations contributed to a change in United Kingdom national immunization policy, with the implementation of the quadrivalent ACWY conjugate vaccine in teenage populations.

Gene-by-gene analysis of WGS data also allows the prevalence of particular vaccine antigens to be determined in any given data set, identifying diversity in these antigens and describing their distribution, which can provide an indication of likely vaccine coverage (37). This is particularly relevant given the fact that a capsular vaccine targeting serogroup B meningococci is unavailable. Consequently, alternative vaccine development approaches have been adopted based on cocktails of protein antigens (38).

Surveillance of N. gonorrhoeae antimicrobial resistance (AMR) is becoming increasingly important, given that some gonococci now exhibit resistance to multiple compounds. The development of genetic methods for the determination of AMR in N. gonorrhoeae is thus critical, particularly since the use of nucleic acid amplification tests (NAATS) to diagnose gonorrhoeae has been rapidly replacing culture methods. Several WGS studies undertaken in N. gonorrhoeae have identified associations between cefixime resistance and possession of *penA* mosaic alleles (39, 40), indicating that determination of antimicrobial resistance patterns from WGS data is achievable and likely to become essential in combatting AMR. All genes implicated in gonococcal AMR have been catalogued into a gonococcal AMR scheme within the pubmlst.org/neisseria website. This enables gene-by-gene analysis of antimicrobial resistance in multiple gonococcal populations in combination with other analyses, including wgMLST. Such analyses identified an association between possession of the gonococcal genetic island (GGI) and resistance to several antimicrobial compounds (O. Harrison, unpublished data). The GGI, a type IV secretion system, significantly increases HGT in gonococci through the secretion of single-stranded DNA in the environment: it is therefore likely that the GGI facilitates the spread of AMR in gonococcal populations.

## CONCLUSIONS

The need for uniform approaches for the characterization of bacterial isolates is exemplified by the Gram stain, developed in 1884 by the Danish bacteriologist Hans Christian Gram; however, this remains the only phenotypic test employed nearly universally on bacterial clinical isolates. This is a consequence of the information content that it generates, its relative simplicity, and its widespread availability. Arguably, the only other laboratory test with such wide applicability is 16S rRNA gene sequencing, developed around 100 years later by Carl Woese, although this is still far from universally available. Recent developments in WGS technology and analysis approaches provide the tantalizing prospect of obtaining entire bacterial sequences for routine purposes, leading to, for example, an improved public health response with the potential for several of the sequencing technologies currently in development to be deployed widely, including in resource-poor settings. The combination of WGS data with hierarchical data analysis approaches, such as those outlined here, will result in high-resolution, universal bacterial characterization methods that will have profound implications in all areas of microbiology. There is now the exciting prospect of using these tools to examine pathogens in the context of the microbial communities that form the microbiome so that the use of analyses devoted to the characterization of individual bacterial isolates, which began with Koch, may now be coming to an end.
